# Modeling multiphase fluid flow, mass transfer, and chemical reactions in bioreactors using large‐eddy simulation

**DOI:** 10.1002/elsc.202200020

**Published:** 2022-11-11

**Authors:** Navraj Hanspal, Brian DeVincentis, John A. Thomas

**Affiliations:** ^1^ Corteva Agriscience Midland Michigan USA; ^2^ M‐Star Simulations Ellicott City Maryland USA

**Keywords:** bioreactor, CFD, mass transfer, multiphase

## Abstract

We present a transient large eddy simulation (LES) modeling approach for simulating the interlinked physics describing free surface hydrodynamics, multiphase mixing, reaction kinetics, and mass transport in bioreactor systems. Presented case‐studies include non‐reacting and reacting bioreactor systems, modeled through the inclusion of uniform reaction rates and more complex biochemical reactions described using Contois type kinetics. It is shown that the presence of reactions can result in a non‐uniform spatially varying species concentration field, the magnitude and extent of which is directly related to the reaction rates and the underlying variations in the local volumetric mass transfer coefficient.

**Abbreviations**
CFD: computational fluid dynamics
GPU: graphics processing unit
LBM: Lattice Boltzmann method
LES: large eddy simulation
MRF: moving reference frame
ODE: ordinary differential equation
RANS: Reynolds averaged Navier Stokes
SLPM: standard liters per minute

## 1 INTRODUCTION

Due to an increased demand for agrochemicals, biofuels, nutrition ingredients, and healthcare products worldwide, the number and scale of industrial bioprocesses continues to expand at a rapid rate [1]. This growth is accompanied by increasing needs for industrial‐scale bioprocesses, complemented with a demand for large‐scale and highly efficient bioreactors. However, one major challenge encountered during process development is the scale‐up and transfer from the lab to industrial scale bioreactors, which may result in reduced biomass and product‐substrate yield, eventually hampering the overall productivity. This reduction in performance in industrial‐scale processes is mostly associated with an inefficient scale‐up of mass, heat transfer and biochemical reactions in addition to mixing issues. Understanding these scale‐up mechanisms for reducing performance losses is extremely critical for successful industrial applications.

Physics‐based modeling approaches, such as computational fluid dynamics (CFD), are important tools in understanding the physics driving mass transfer in bioreactors. For example, Lapin et al. pioneered the idea of tracking single cell lifelines within an Euler‐Lagrange framework to evaluate the performance of industrial scale bioreactors [2, 3]. They used CFD to evaluate the dynamic behavior of yeast cells and the effect of glucose gradients on the metabolic response with simplified glucose uptake kinetics. Although numerous other CFD based‐reaction engineering studies have been reported in the literature [(4), (5), (6)], a majority of them have significantly relied on the utilization of conventional RANS and MRF type approaches coupled with reaction kinetics. Furthermore, a majority of conventional and commercial CFD solvers present limited compatibility with high‐performance computing architectures such as Graphics Processing Units (GPUs) [7] thereby, severely restricting their applicability to bioreactor simulations involving transient computations with prolonged time‐scales. Alternatively, the lattice‐Boltzmann method (LBM) has high parallelism and explicit time‐marching characteristics, rendering it to be a suitable tool for transient computations [8].

In this work, we present a large‐eddy‐simulation (LES) based approach to simulating a bioreactor system based on resolution of the transient flow field, which can inherently capture the time fluctuations of the species concentrations, subject to imperfect mixing, that can alter the overall yield and productivity of the system. Another focus of this study is to explore the feasibility of coupling a lattice‐Boltzmann based Euler‐Lagrange CFD model in a transient framework with unstructured kinetics describing the gluconic acid production by *Aspergillus Niger* in an aerobic fermentation process. [9] For modeling biochemical reactions, these published kinetic parameters have been utilized whereby the gluconic acid fermentation is described by a Contois kinetics model.

We begin in Section 2 by discussing the mathematical models governing transport within the bioreactor. Next, in Section 3, various aspects related to theoretical model validation, considering a range of simulation scenarios involving no‐reactions, imposition of uniform reaction rates and finally integrating Contois type reaction kinetics with the additional bioreactor physics have been illustrated. In Sections 3.4 and 3.5, the practicality of the developed CFD approach has been discussed in context of optimizing the gluconic acid production through modeling based, simulation studies. Finally, we list the conclusions in Section 4 with an aim to expand the applicability of this developed approach to industrial bioreaction systems in large‐scale vessels. The presented approach can be used to model and predict hours/days of process mechanics, providing wide spectrum insights into spatial‐temporal variations in process behavior occurring within the system.

## 2 THEORETICAL CONSIDERATIONS

### 2.1 Governing equations

Our aim is to develop a generalized bioreactor model based on first principles conservation laws. As a foundation, we model fluid flow and species transport by solving the transient Navier‐Stokes equations in tandem with the transient advection‐diffusion‐reaction Equations (10) and (11):

(1) $$\frac{\partial \vec{V}}{\partial t}+\vec{V}\cdot \nabla \vec{V}=g-\frac{\nabla p}{\rho }+\nabla \cdot \left(\nu \nabla \vec{V}\right),$$

and

(2) $$\frac{dc_{j}}{\mathrm{dt}}=\nabla \cdot \left(D_{j}\nabla c_{j}\right)-\nabla \cdot \left(\vec{V}c_{j}\right)+\frac{{\dot{n}}_{j}}{V}+R_{j}$$

Within Equation (1), which represents the conservation of fluid momentum, $\vec{V}$ is the fluid velocity, *t* is time, *g* is gravity, *p* is pressure, ρ is density, and ν is the kinematic viscosity. Within Equation (2), which represents the conservation of mass, $c_{j}$ is the concentration of species *j*, *D_j_* is the species diffusion coefficient, ${\dot{n}}_{j}$ is the mass transfer rate within volume *V* and $R_{j}$ is the reaction rate. Although we assume that the fluid is incompressible (divergence‐free), the viscosity is allowed to be non‐uniform. Turbulence is handled using the Lilly‐Smagorinsky eddy viscosity closure model with a Smagorinsky coefficient of 0.1. [12] Since these two governing equations are solved in tandem, changes in the species concentration can inform the local viscosity. We solve Equations (1) and (2) using the lattice‐Boltzmann (LB) method (13), as discussed in detail in our previous reports ((14), (15), (16), (17)).

Bubbles are modeled as discrete spherical objects that are tracked individually. The motion of each bubble *i* is described using Newton's second law:

(3) $$m_{i}{\vec{a}}_{i}={\vec{F}}_{i,D}+{\vec{F}}_{i,p}+{\vec{F}}_{i,a}+{\vec{F}}_{i,g}$$

where $m_{i}$ and ${\vec{a}}_{i}$ represent the mass and acceleration vectors, ${\vec{F}}_{i,D}$ is the drag force, ${\vec{F}}_{i,p}$ is the pressure gradient force, ${\vec{F}}_{i,a}$ is the added mass, and ${\vec{F}}_{i,g}$ is the combined force due to gravity and buoyancy. Equation (3) is solved for each bubble and in tandem with the fluid algorithm using the velocity Verlet algorithm [18]. Bubble forces are (two‐way) coupled via Newton's third law to the fluid through drag, density and added mass [19]. Bubble break‐up and coalescence are handled at the level of individual bubbles and bubble pairs, as discussed in detail in our previous reports [(15), (16), (17)].

For each species, mass transfer between each bubble and the surrounding liquid is modeled as:

(4) $${\dot{n}}_{i,j}=k_{L,i,j}A_{i}\left(S_{j}c_{g,i,j}-c_{f,i,j}\right)$$

where ${\dot{n}}_{i,j}$ is the rate of mass transfer of species *j* across the bubble interface, $k_{L,i,j}$ is the species‐specific mass transfer coefficient in the fluid surrounding the bubble, $A_{i}$ is the surface area of the bubble, $S_{j}$ represents the species‐specific dimensionless solubility, $c_{g,i,j}$ is the gas‐phase species concentration, and $c_{f,i,j}$ is the dissolved species concentration in the fluid surrounding the bubble. The bubble volume is coupled to mass flux, such that ongoing species exchange to/from the fluid causes the bubble diameter to change in time [(15), (16)].

The mass transfer coefficient of the fluid around each bubble is calculated from Equation (20):

(5) $$k_{L,i,j}=C{\left(\varepsilon_{i}\nu_{i}\right)}^{1/4}Sc_{i,j}^{\beta }$$

where $Sc_{ij}$ is the species‐specific Schmidt number in the fluid around the bubble. The constants *β* and *C*, which are defined from turbulence theory, are set to ‐½ and 0.301 Equation (21).

We reemphasize that the transport processes within the simulation are modeled at the level of individual bubbles moving through individual fluid lattice sites. If a system with no reactions is well‐stirred and the gas concentration in the bubbles is assumed to be constant, Equations (2) and (4) can be simplified into:

(6) $$\frac{dc_{l}}{dt}=\frac{k_{l}A\left(Sc_{b}-c_{l}\right)}{V_{l}}=k_{l}a\left(Sc_{b}-c_{l}\right)$$

where $c_{b}$ represents the gas concentration of the bubbles. In this form, $k_{l}$ represents the total/effective mass transfer coefficient of all bubbles in the system, *A* is the total bubble surface area, and $V_{l}$ is the total liquid volume (prior to gassing). In the right‐hand expression, *a* is the specific surface area of the bubbles in the vessel. The factor $k_{l}a$ is named the volumetric mass transfer coefficient and has units of reciprocal time [20]. Note that $1/k_{l}a$ is a parameter that has units of time and describes a characteristic time scale required for the mass transfer process to equilibrate.

### 2.2 Model integration

**FIGURE 1:**
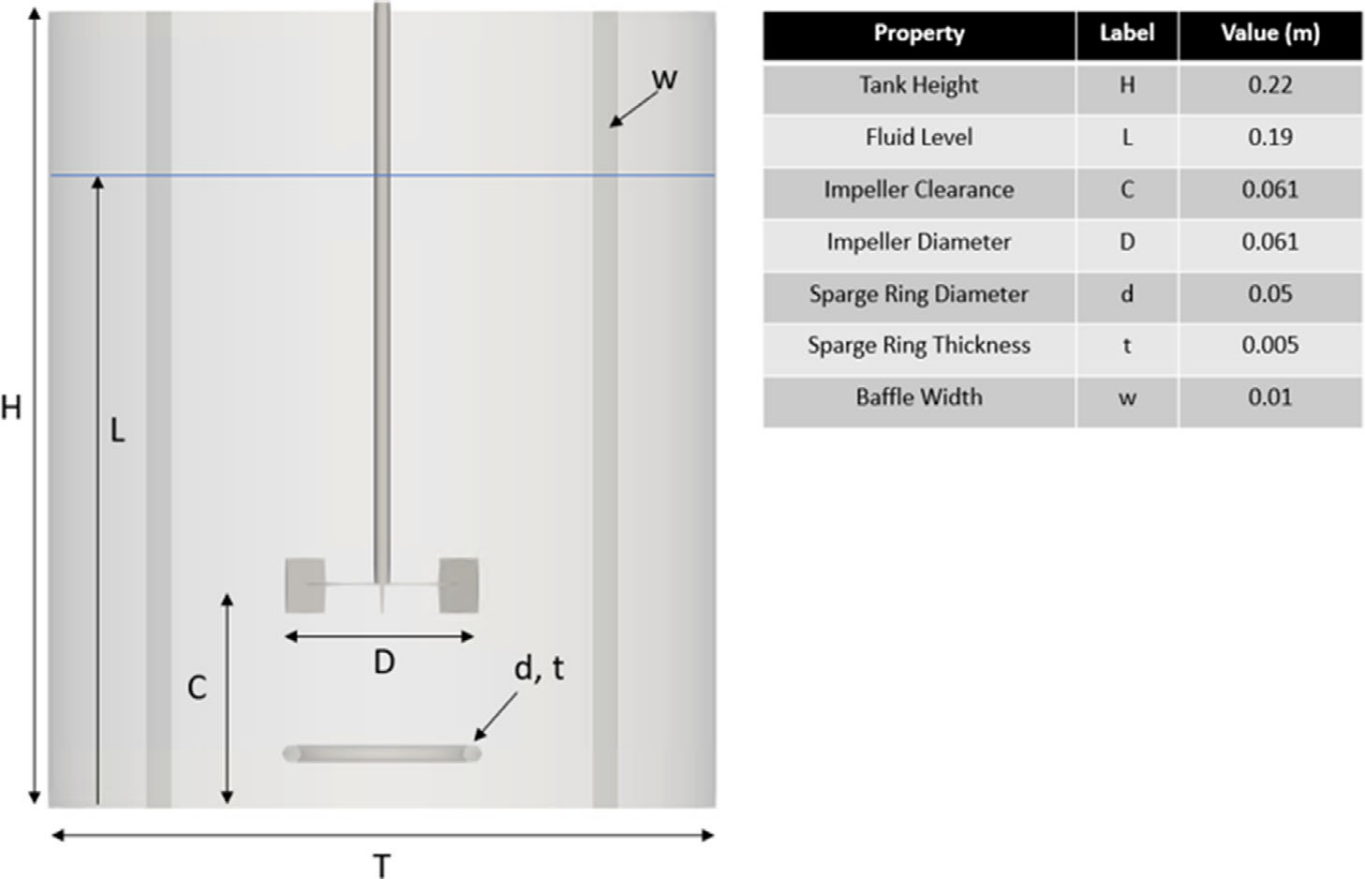
Key geometric parameters of the bioreactor model considered in this work. The initial height of the free surface interface corresponds to a fluid volume of 3.9 L. When gassed, bubbles entered the tank through holes along the ring sparge.

In Figure 1, we present a snapshot of the system topology considered in this work. The vessel, patterned after the work of Elqotbi et al. [22], is a baffled 0.185 m diameter flat‐bottomed cylindrical tank with four 0.01 m baffles. The fluid was agitated by a 0.061 m diameter Rushton impeller that was centered in the vessel with an off‐bottom clearance equal to the impeller diameter. A 0.05 m ring sparge was centered in the tank and positioned between the impeller and the tank bottom. The initial liquid level was set to 0.185 m. The impeller speed varied between runs, as discussed below.

Within the context of the LB simulation, interactions between the fluid and the tank walls/ring sparge were modeled using a no‐slip bounce‐back boundary condition [23]. Interactions between the fluid and the moving impeller were treated using the immersed boundary method [13]. Free surface dynamics were modeled using the volume‐of‐fluid (VOF) approach [24]. Bubbles entered along the top surface of the sparge ring per a user‐defined size distribution. The displacing effects of the added gas are modeled explicitly, meaning the free surface level will rise with increasing gas hold‐up.

**FIGURE 2:**
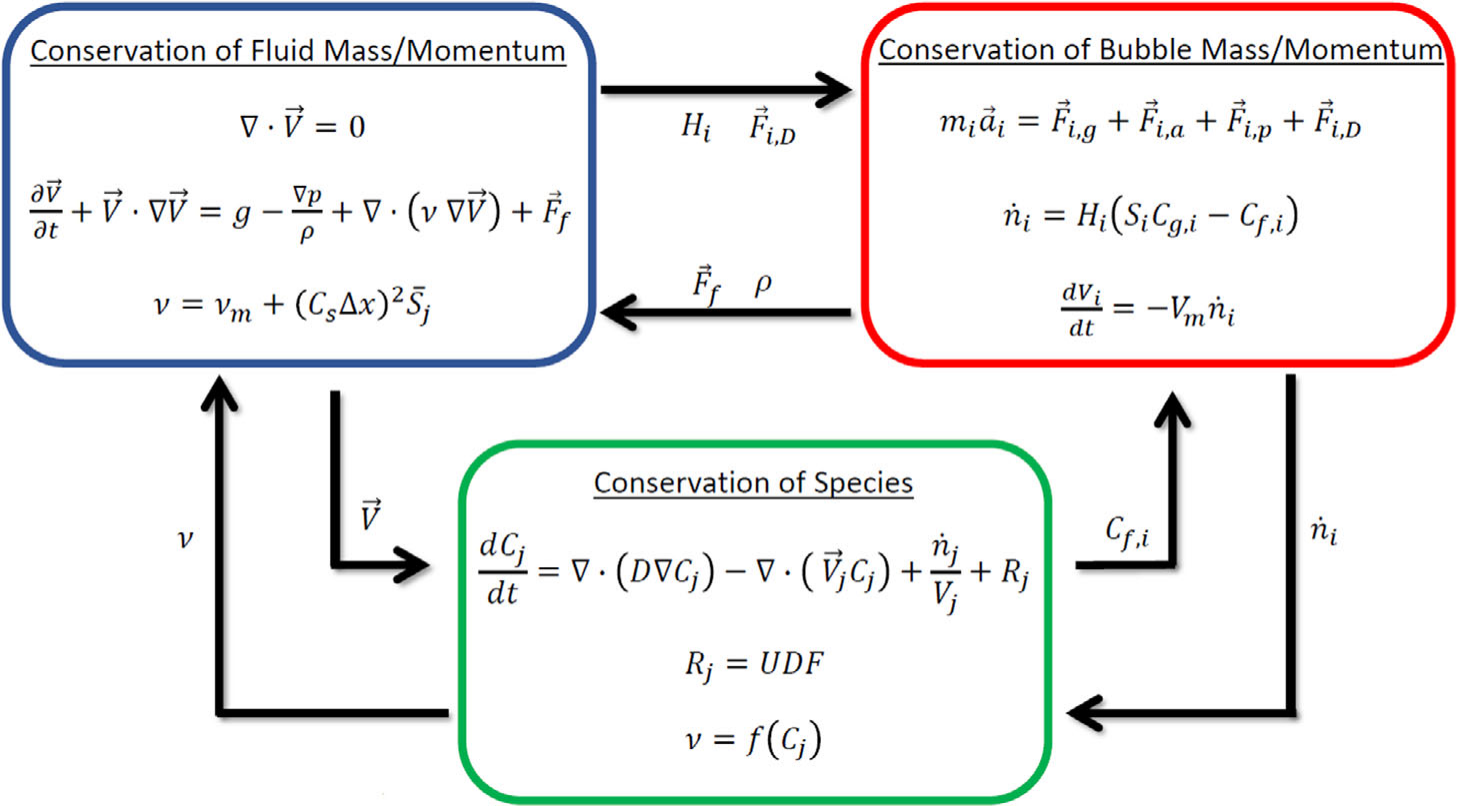
Flow chart illustrating the interactions between fluid flow, bubble transport, and species transport. All physics are solved at each lattice site using the same timestep

In Figure 2, we summarize the mathematical coupling between fluid flow, bubble transport, and species transport. Beginning in the top left corner, as discussed above, incompressible fluid mechanics are governed by the Navier‐Stokes equation with a LES closure model. The instantaneous fluid viscosity at each lattice point is informed by (1) the local species concentrations and (2) the effects of the LES filter. Per Newton's third law, the instantaneous body force on each lattice site is informed by the bubble density, added mass, and drag force.

Moving to the top right corner, the velocity and flow field predicted by solving the Navier‐Stokes equations informs both bubble transport and the mass flux between phases across the bubble interface. More specifically, the bubble drag force is a function of the slip velocity, while the mass transfer coefficient around each bubble depends on the local fluid velocity gradient (via the energy dissipation rate). This transfer coefficient, combined with the concentration differences between the gas bubbles and the surrounding fluid, then informs mass transfer between each bubble and the local fluid volume.

In the bottom box, mass transfer is governed by the advection‐diffusion‐reaction equation. Fluxes between lattice sites are informed by the local fluid velocity, and source/sink terms are informed by exchange with the bubbles. Superimposed on these transport mechanics are any user‐defined functions (UDF) for the underlying consumption/production reactions. Changes in the local species concentration can modify the viscosity at each lattice site, which informs the solution of the Navier‐Stokes equations. The full coupling of these physics is considered for each lattice site at each simulation time step.

### 2.3 Grid spacing and timestep considerations

The LB algorithm is an explicit time‐marching approach that is second‐order accurate [25]. The approach is conservative to machine precision and all interactions are localized in space. To set up a simulation, a lattice spacing Δx and simulation time step Δt must be user‐defined. The lattice spacing is uniform in the models considered here. This lattice spacing, also called the grid resolution, informs the smallest eddies and hydrodynamic length scales that will be explicitly captured by the simulation. Since the algorithm is explicit, the time step defines the lattice velocity and the compressibility of the modeled fluid. As discussed in previous work, we apply a uniform grid‐spacing of 100 points across the impeller diameter – a value sufficient to realize converged and grid‐independent power draw and blend time predictions [14].

The simulation Δt is defined using the lattice resolution and reference velocity, $V_{r}$:

(7) $$\Delta t=\frac{Co\Delta x}{V_{r}}$$

where Co is the simulation Courant number. We set $V_{r}$ to be the maximum tip speed of the impeller. The Courant‐Friedrichs‐Lewy condition requires Co to be less than 1.0, and we choose a value of 0.1 for this work. This value, which was motivated by previous simulations, ensures that lattice density fluctuations across the fluid remain below a 2% [14].

## 3 MODELING MASS TRANSFER

### 3.1 Oxygen saturation with no reaction

As a first application of the modeling framework, we examine the saturation of dissolved oxygen in a lab‐scale bioreactor. Pure oxygen enters the system at 8 SLPM as discrete bubbles through the ring sparge below the impeller. The injected gas bubble diameter was 2 mm [26]. The density and corresponding molar volume of the added bubbles were set to 1.2 kg/m^3^ and 22.4 L/mol. The dissolved oxygen diffusion coefficient was set to 10^−9^ m^2^/s, which is representative of notional gas‐liquid diffusion coefficients [27]. For the dissolved oxygen, we set the dimensionless solubility to be 0.032.

**FIGURE 3:**
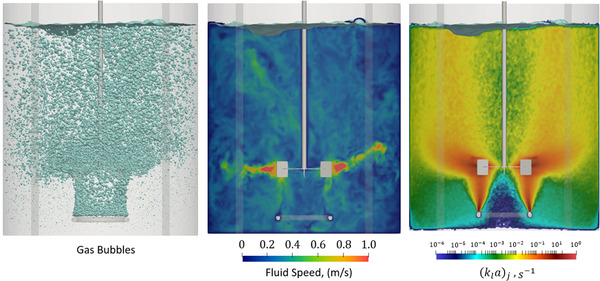
Left: Snapshot of the bubble field after 50 s of agitation. The bubble number density and surface area are highest near the sparge and within the trailing vortex. Center: Snapshot of the fluid velocity along a center plane through the tank center. Right: Variations in the time‐average volumetric mass transfer coefficient, $\left\langle k_{l}a\right\rangle$, along the center plane.

In Figure 3, we show a snapshot of the simulation output after 50 s of agitation and sparging. In the left pane, we present the three‐dimensional bubble distribution across the tank. By this point, the fluid flow, bubble count, and bubble size distribution have established a quasi‐steady state with no long‐term variations in their mean values. The gas hold‐up for this system is around 5%. The bubble number density and surface area are maximized in the trailing vortex surrounding the impeller. Away from the impeller, the effects of coalescence cause the bubbles to become larger as they approach the free surface.

In the center pane, we present the fluid velocity along a plane crossing through the impeller shaft. The fluid satisfies the no‐slip boundary condition near the impeller and at the tank wall. The maximum fluid velocity is consistent with the impeller tip speed of 0.96 m/s. A small vortex moves along the free surface interface, although the effect of this vortex on fluid flow appears negligible.

In the right pane, we present the spatial variations in the time‐averaged volumetric mass transfer coefficient, [$k_{l}a$], which is calculated from:

(8) $$k_{l}a=\frac{1}{\tau }\underset{0}{\int^{\tau }}\frac{k_{l}\left(t\right)A_{T,j}\left(t\right)}{V_{j}}\mathrm{dt}$$

In Equation (8), $k_{l}\left(t\right)$ and $A_{T}\left(t\right)$ are the instantaneous mass transfer coefficient and bubble surface area at each lattice site, $V_{j}$ is the lattice site volume, and τ represents an averaging window [15]. The mass transfer coefficient shows very strong spatial variations across the tank. It is maximized near the impeller, where the bubble surface area and $k_{l}$ are both high. Near the wall, where coalescence leads to a lower bubble specific surface area, the fluid has lower energy dissipation rates and the mass transfer coefficient is depressed by multiple orders‐of‐magnitude. These spatial variations will have a direct influence on species concentration differences in reacting systems, a point we detail in the next section.

**FIGURE 4:**
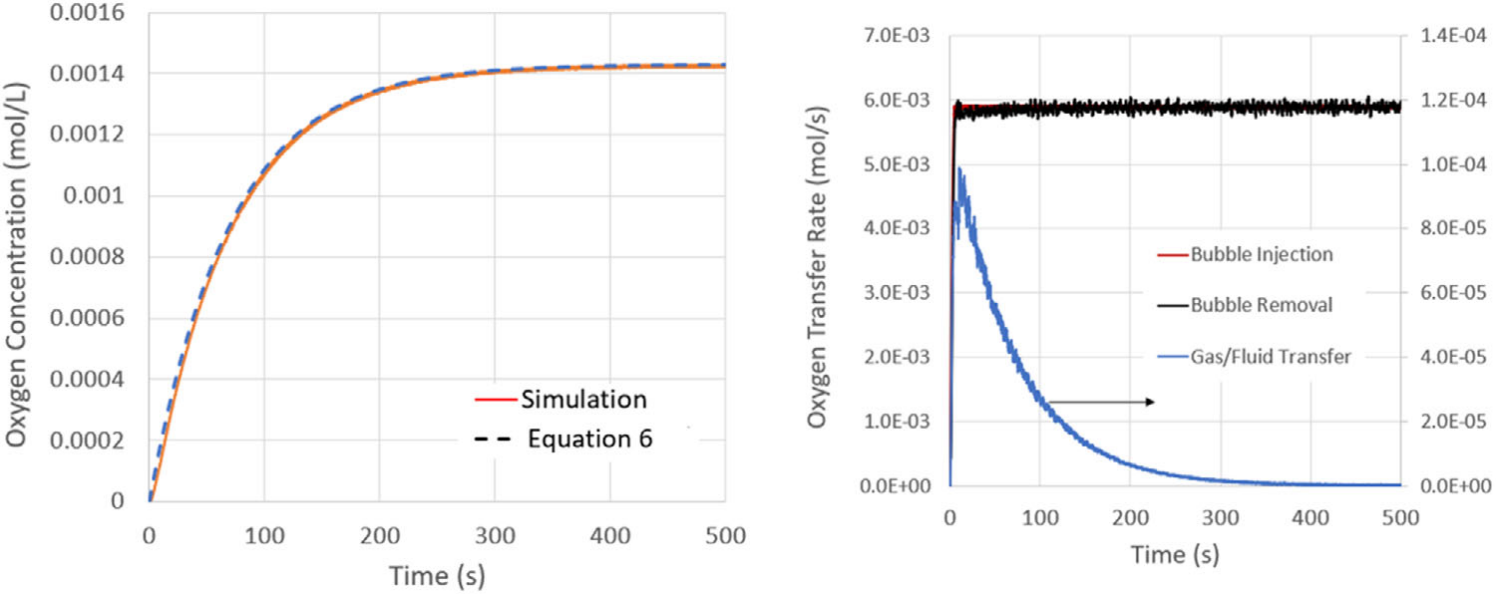
Left: Evolution of the mean dissolved oxygen concentration. With increasing time, the dissolved oxygen concentration converges to the saturation limit of $Sc_{b}=0.00143\mathrm{mol}/L$. Superimposed on this data is the solution to Equation (6), with a best fit value of $k_{l}a=0.014s^{-1}$ Right: Oxygen flux into the vessel (contained in bubbles entering through the sparge), oxygen flux leaving the vessel (contained in bubbles leaving the free surface), oxygen flux into the fluid (moving across the bubble/liquid interface).

In Figure 4, we present the evolution of the dissolved oxygen concentration in the vessel. With increasing time, as expected, the dissolved oxygen concentration evolves from zero to a steady‐state value of 0.0143 mol/L. This steady‐state concentration corresponds to the product of the specific molar volume of the gaseous oxygen entering the system (1/22.4 mol/L) and the liquid phase solubility. The effective volumetric mass transfer coefficient, $k_{l}a$, is calculated to be 0.014 1/s by fitting this data to Equation (6). This value agrees with other experimental and simulation predictions at similar operating conditions [9, 22].

To understand the mass transfer processes driving this behavior, we also plot in Figure 4 the molar flux of oxygen to the fluid and entering/leaving the system via the bubbles. Within this plot, the “Gas/Fluid Transfer” rate represents the molar flux of oxygen from the bubbles to the liquid. The “Bubble Injection” rate is the rate at which oxygen enters the system contained within gas bubbles via the sparge. The “Bubble Removal” rate is the rate at which oxygen exits the system contained within gas bubbles via the free surface. As expected in the long‐time limit, when the fluid becomes oxygen saturated, all oxygen that enters the system (via the gas bubbles at the sparge) leaves the system in gas bubbles exiting the free surface. The Gas/Fluid transfer rate at this time is zero and the Injection/Remove rates are identical. Prior to reaching this equilibrium state, the Gas/Fluid transfer rate is positive as oxygen accumulates in the fluid. Consequently, more oxygen enters the system within the gas bubbles via the sparge than exits the system within gas bubbles via the free surface. During the period of peak mass transfer (i.e., the first 60 s of sparging) no more than 2% of oxygen flowing into the system via the sparge was transferred to the liquid. That is, the utility of the oxygen entering through the sparge is low over the duration of the mass transfer process.

### 3.2 Oxygen saturation with reaction

Encouraged by the self‐consistency of the oxygen saturation modeling, we now superimpose an oxygen consumption/carbon dioxide production reaction into the mass transfer processes. Within the context of Equation (2), the corresponding consumption and production reactions become:

(9) $$R_{O_{2},j}=-10^{-5}\frac{mol/L}{s}$$

(10) $$R_{CO_{2},j}=10^{-5}\frac{mol/L}{s}$$

such that oxygen is consumed in the fluid at the same rate at which carbon dioxide is produced. These reactions are purposely simple and only consider the production of non‐organic carbon dioxide (which can be transferred to the bubbles). As will be discussed in Section 3.3, the reaction terms can be further generalized to handle multi‐species/coupled competition reactions. The reactions are solved locally for each lattice site at each simulation timestep. The dissolved carbon dioxide diffusion coefficient was taken to be 10^−9^ m^2^/s, a value representative of dissolved gases [27]. The dimensionless solubility of the carbon dioxide was set to 0.83 [28]. No dissolved carbon dioxide was initially present in the liquid prior to gassing. The density and molar volume of the gaseous carbon dioxide, when contained within the bubbles, were taken to be 1.2 kg/m^3^ and 22.4 L/mol. The properties of the oxygen are the same as those applied in Section 3.1.

Even this simplified consumption/production reaction has three important implications on mass transfer. First, in addition to transferring oxygen to the fluid, bubbles will now also act to strip inorganic carbon dioxide from the fluid and remove it from the system. Thus, the composition of individual bubbles (which still enter as pure oxygen) will change over time as they accumulate carbon dioxide. Second, within the fluid, the concentration of dissolved carbon dioxide will increase over time until the mass transfer rate to the bubbles becomes equal to the carbon dioxide production rate within the fluid. From Equation (6), if the bubbles remain mostly oxygen, the carbon dioxide concentration at this equilibrium condition can be estimated as:

(11) $$c_{o,CO_{2}}=\frac{R_{CO_{2}}}{{\left(k_{l}a\right)}_{CO_{2}}}$$

where $c_{o,CO_{2}}$is the steady‐state average dissolved carbon dioxide concentration in the fluid, $R_{CO_{2}}$ is the non‐organic carbon dioxide production rate, and ${\left(k_{l}a\right)}_{CO_{2}}$ is the volumetric mass transfer coefficient of carbon dioxide.

The third key effect of the consumption/production reaction is to decrease, relative to the non‐reacting system, the tank‐average steady‐state dissolved oxygen concentration. For reacting systems, the concentration of dissolved oxygen will increase over time until the mass transfer rate from the bubbles becomes equal to the oxygen consumption rate within the fluid. From Equation (6), if the bubbles remain mostly oxygen, the dissolved oxygen concentration that corresponds to this equilibrium condition can be estimated as:

(12) $$c_{o,O_{2}}=S_{O_{2}}c_{b,O_{2}}-\frac{R_{O_{2}}}{{\left(k_{l}a\right)}_{O_{2}}}$$

where $c_{o,O_{2}}$ is the equilibrium concentration of dissolved oxygen, $S_{O_{2}}$ is the dimensionless solubility of dissolved oxygen, $c_{b,O_{2}}$ is the oxygen concentration in the bubbles, $R_{j,O_{2}}$ is the dissolved oxygen consumption rate, and ${\left(k_{l}a\right)}_{O_{2}}$ is the volumetric mass transfer coefficient of oxygen.

In many biochemical systems, the mass transfer rate is fast relative to the chemical reaction rates. For such systems, we can use Equation (6) to define:

(13) $$c_{o,O_{2}}=S_{O_{2}}c_{b,O_{2}}\left(1-\xi \right)$$

where ξ is the ratio of oxygen consumption to oxygen transfer:

(14) $$\xi =\frac{R_{O_{2}}}{S_{O_{2}}c_{b,O_{2}}{\left(k_{l}a\right)}_{O_{2}}}.$$

This dimensionless ratio, which will be discussed below, provides insight into how bioreactor operating conditions can be optimized to maximize process yield. Recognize that Equation (14) assumes that the dissolved oxygen concentration is below the saturation limit.

**FIGURE 5:**
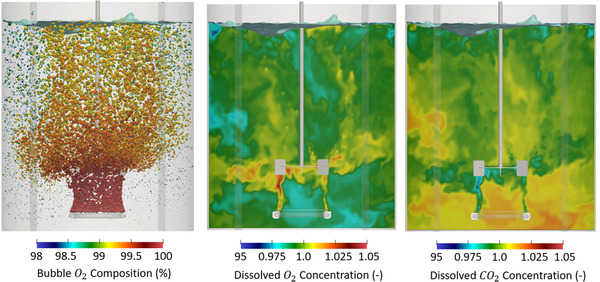
Left: Snapshot of the bubble oxygen composition after 600 s of agitation (steady‐state flow conditions). The bubbles enter as pure oxygen and see little variation in composition as they move through the tank. Center: Image of the dissolved oxygen concentration along the center plane. The dissolved oxygen concentration is normalized by 0.00065mol/L, which is the steady‐state tank‐average dissolved oxygen concentration. Right: Snapshot of the dissolved carbon dioxide concentration. The dissolved carbon dioxide concentration is normalized by 0.00096mol/L, which is the steady‐state tank‐average dissolved carbon dioxide concentration.

With these three key effects as background, in Figure 5 we present an image of the bubble oxygen composition, dissolved oxygen concentration, and dissolved carbon dioxide concentration across the tank after 600 s of agitation. By this time, the system has reached steady‐state operating conditions, in terms of total dissolved oxygen and dissolved carbon dioxide concentrations. As discussed above, although bubbles enter the system as pure oxygen, they accumulate carbon dioxide as they move through the fluid. Since the bubble residence time (which is on the order of seconds for this system) is small relative to the mass transfer time scales, this accumulation results in only a 1%–2% change in the gaseous composition of each bubble. In systems with longer bubble residence times or higher mass transfer rates, this decrease is expected to be more substantial.

In contrast to the non‐reacting case, which converges to a uniform concentration equal to the saturation limit, the on‐going oxygen consumption causes the dissolved oxygen concentration to vary across the fluid. Since the system is well stirred, these variations are only on the order of +/‐ 5% relative to the mean dissolved oxygen concentration. The mechanisms driving this behavior, can be understood from Equation (14): for a constant reaction rate and bubble concentration, the local dissolved oxygen concentration is expected to increase with increasing local $k_{l}a$. Accordingly, in regions where the mass transfer coefficients are elevated, the dissolved oxygen concentration will be high. Outside the trailing vortex, where the mass transfer coefficient is lower, the local dissolved oxygen concentration is low. The dissolved carbon dioxide concentration likewise varies +/‐ 5% across the fluid, with the lowest concentrations near the impeller and the highest concentrations near the bottom of the tank. This behavior is consistent with Equation (15), which suggests that the dissolved carbon dioxide concentration will be low in regions where local $k_{l}a$ is high. The key point is that ongoing reactions can produce spatially varying species concentrations across the fluid.

**FIGURE 6:**
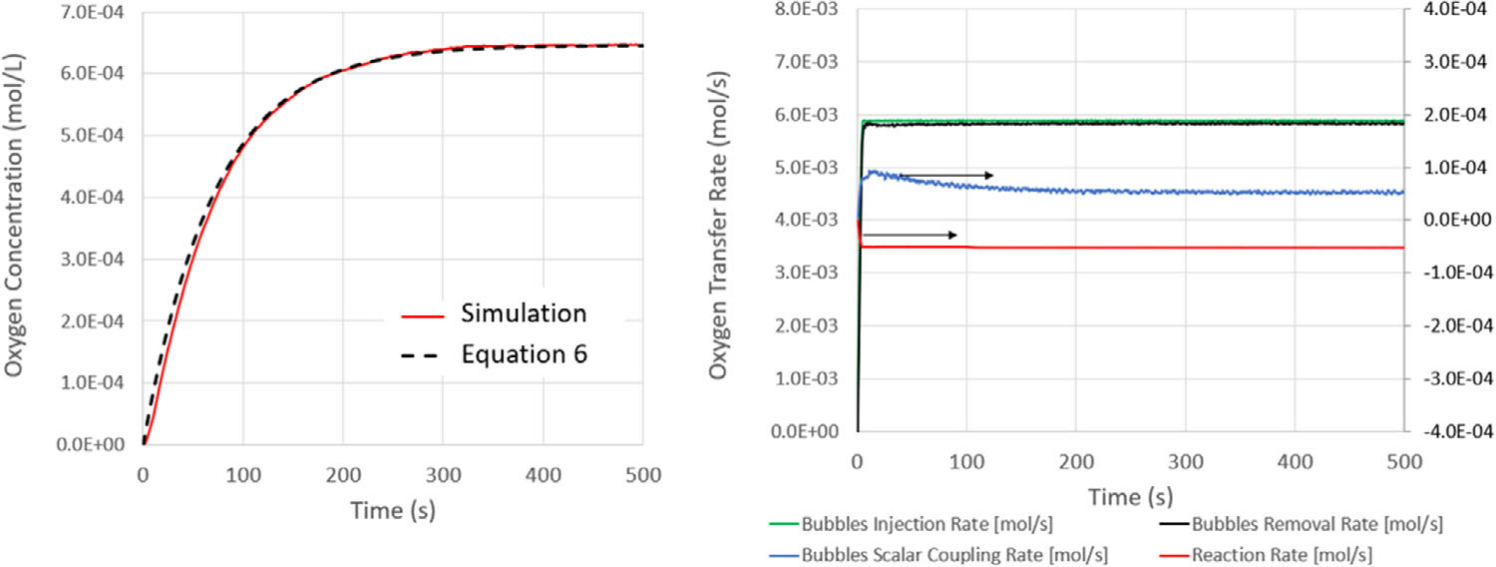
Left: Evolution of the mean dissolved oxygen concentration, with ongoing oxygen consumption. The dissolved oxygen concentration converges to a steady‐state value of 0.00065mol/L. Superimposed on this data is the solution to Equation (6), with a best fit value of $k_{l}a=0.014s^{-1}$ Right: Oxygen flux into the vessel (contained in bubbles entering through the sparge), oxygen flux leaving the vessel (contained in bubbles leaving the free surface), oxygen flux into the fluid (moving across the bubble/liquid interface), and oxygen consumption within the fluid by the reaction.

To further understand the competition between oxygen transfer and oxygen consumption, in Figure 6 we plot the time‐evolution of the dissolved oxygen concentration. We fit this data to Equation (6) to predict a ${\left(k_{l}a\right)}_{O_{2}}$ value of 0.014 1/s. This prediction is in‐line with the non‐reacting case. The agreement between the two predictions is expected since the dissolved carbon dioxide has no effect on the oxygen mass transfer coefficient or bubble break‐up kinetics within the model. The dissolved oxygen concentration converges to 0.00064 mol/L – a value consistent with a priori predictions from Equation (14).

We also plot in Figure 6 the molar flux of oxygen entering/leaving the system via the bubbles and entering/leaving the fluid through transfer and reaction. Within this plot, the “Gas/Fluid Transfer” rate represents the molar flow rate of oxygen from the bubbles to the liquid. The “Reaction” rate represents the rate at which oxygen is consumed by the reaction within the fluid. The “Bubble Injection” rate is the molar flow rate at which oxygen enters the system within gas bubbles via the sparge. The “Bubble Removal” rate is the molar flow rate at which oxygen exits the system within gas bubbles via the free surface.

Recognize that the “Bubble Injection” rate is constant since the gas flow rate into the vessel through the sparge is maintained at 8 SLPM. Likewise, after a few seconds start‐up/equilibration period, the “Bubble Removal” rate becomes constant and achieves a value slightly below the “Bubble Injection” rate. The difference between the “Bubble Injection” and “Bubble Removal” rate is equal to the “Gas/Fluid Transfer” rate. That is, as expected from the conservation of mass, any oxygen that enters through the sparge but does not leave through the free surface must have been transferred to the fluid.

Unlike the non‐reacting case, the “Gas/Fluid Transfer” rate does not approach zero. Rather, as discussed above, the “Gas/Fluid Transfer” rate converges to the “Reaction” rate as the system realizes an equilibrium mean dissolved oxygen concentration. Despite the ongoing reaction, still only a small fraction (<1%) of the oxygen that enters the system through the sparge is transferred to the fluid and consumed by the reaction. That is, only a small fraction of the oxygen that enters the system ends up participating in the reaction. If the bubble residence times were longer, as would be realized by smaller bubbles, more oxygen would be transferred to the liquid.

**FIGURE 7:**
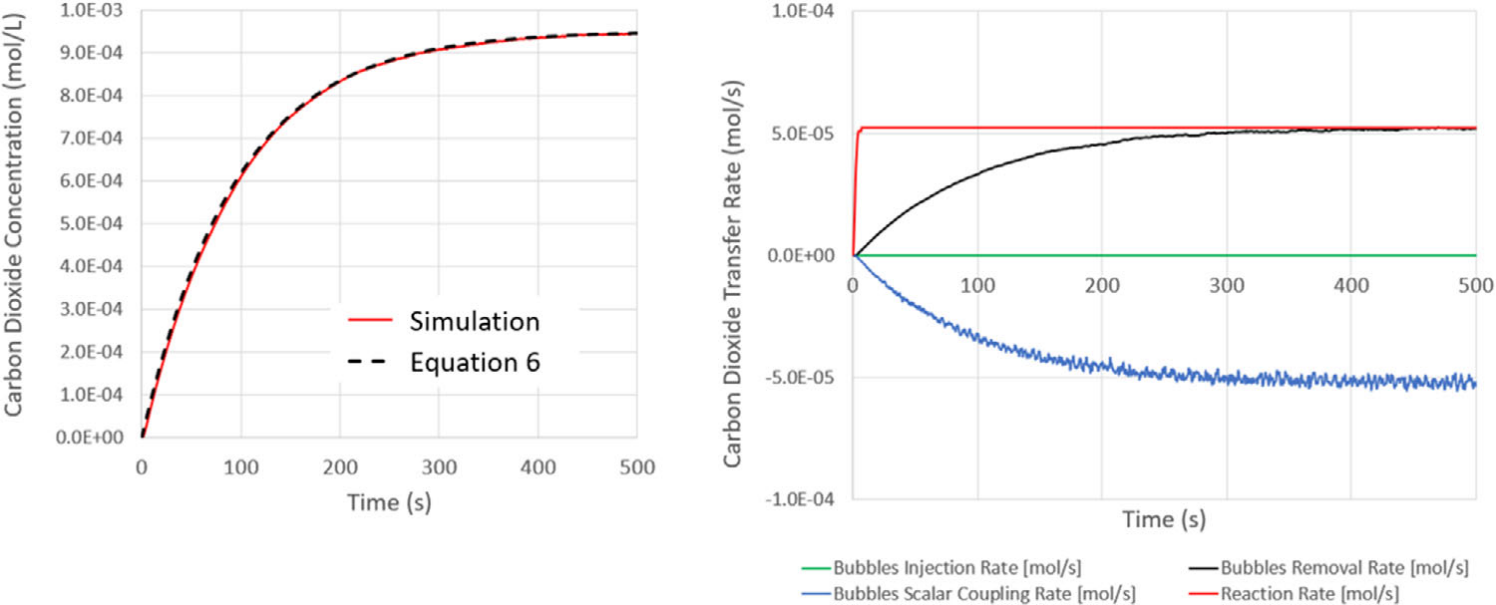
Left: Evolution of the mean dissolved carbon dioxide concentration. With increasing time, the dissolved gas concentration converges to 0.00096mol/L. Superimposed on this data is the solution to Equation (6), with a best fit value of $k_{l}a=0.011s^{-1}$ Right: Carbon dioxide flux into the vessel (contained in bubbles entering through the sparge), flux leaving the vessel (contained in bubbles leaving the free surface), flux into the fluid (moving across the bubble/liquid interface), and consumption by the reaction.

In Figure 7, we present the evolution of the dissolved carbon dioxide concentration. We fit this data to Equation (6) to predict a ${\left(k_{l}a\right)}_{CO_{2}}$ value of 0.011 1/s and a steady‐state dissolved carbon dioxide concentration of 0.00096 mol/L. This concentration is in line with predictions from Equation (14). To further understand these trends, we also plot in Figure 7 the molar flux of carbon dioxide entering/leaving the system via the bubbles and entering/leaving the fluid through transfer and reaction. Since the bubbles enter the system as pure oxygen, the carbon dioxide “Bubble Injection” rate is zero during the entire simulation. Likewise, following a brief start‐up period, the “Reaction” rate remains constant during the simulation. This behavior is expected since the reaction rate is uniform and constant. The “Bubbles Scalar Coupling” rate and “Bubble Removal” *rates* evolve in time in response to system dynamics. As the dissolved carbon dioxide concentration rises, the “Bubble Scalar Coupling” rate increases until the magnitudes of the “Reaction”, “Bubble Scalar Coupling”, and “Bubble Remove” rates converge. This result is expected from the conservation of species: During steady state operating conditions, the rates at which carbon dioxide is generated in the fluid, transferred to the bubbles, and leaves the system *within the bubbles* will *all* become equal. The mean *dissolved carbon dioxide* concentration converges to the value that realizes this equilibrium.

### 3.3 Coupled reactions and gluconic acid production

In this section, we extend the model to handle multiple species reactions with time‐varying kinetics. We apply the simulation specifically to the production of gluconic acid via the fermentation by *aspergillus niger*. This reaction, as parameterized by Zand et al. [9], is a multi‐species reaction that is a function of biomass concentration *X*, gluconic acid concentration *P*, substrate concentration*S*, and dissolved oxygen concentration *O*_2_:

(15) $$R_{X}=\mu C_{X}$$

(16) $$R_{P}=\left(\alpha \mu +\beta \right)C_{X}$$

(17) $$R_{s}=-\left(\gamma \mu +\lambda \right)C_{X}$$

(18) $$R_{O_{2}}=-\left(\delta \mu +\phi \right)C_{X}$$

where $R_{X}$, $R_{P}$, $R_{s}$, and $R_{O_{2}}$ are the reaction rates of each species. The coefficients α, β, γ, and λ are fitting parameters evaluated using measured reactor data. The growth rate μ is taken here to follow the Contois model, such that:

(19) $$\mu =\mu_{m}\left(\frac{C_{S}}{K_{S}^{\prime }C_{X}+C_{S}}\right)\left(\frac{C_{O_{2}}}{K_{0}^{\prime }C_{X}+C_{O_{2}}}\right)$$

where $C_{S}$ is the substrate concentration and $C_{X}$ is the biomass concentration. The parameters $\mu_{m}$, $K_{S}^{\prime }$ and $K_{0}^{\prime }$ are measured experimentally. The parameters used in this reaction are taken from the literature [9]. Within the simulation, the reactions are solved locally at each lattice site in tandem with the fluid flow using a 4^th^ order Runge–Kutta solver with a timestep equal to the simulation timestep.

The physical and numerical operating conditions modeled here are like those applied in Sections 3.1 and 3.2, although the resolution was decreased to 150 lattice points across the tank diameter to decrease simulation runtimes. This resolution is still sufficient to resolve flow, blending and energy dissipation rates around a Rushton impeller [29]. Also, due to the ongoing reaction, the local viscosity was written to be a function of the local biomass concentration [22]:

(20) $$\nu =\nu_{r}\left(1+C_{X}^{2.26}\right)$$

where ν is the local fluid viscosity, $\nu_{r}$ is the viscosity of water, and $C_{X}$ is the local biomass concentration. This expression is evaluated for each lattice site at each time step when solving Equation (1). The systems here were sparged with air, modeled as 21% oxygen and 79% nitrogen. The initial concentrations of each species were uniformly distributed across the fluid at the beginning of the simulation.

**FIGURE 8:**
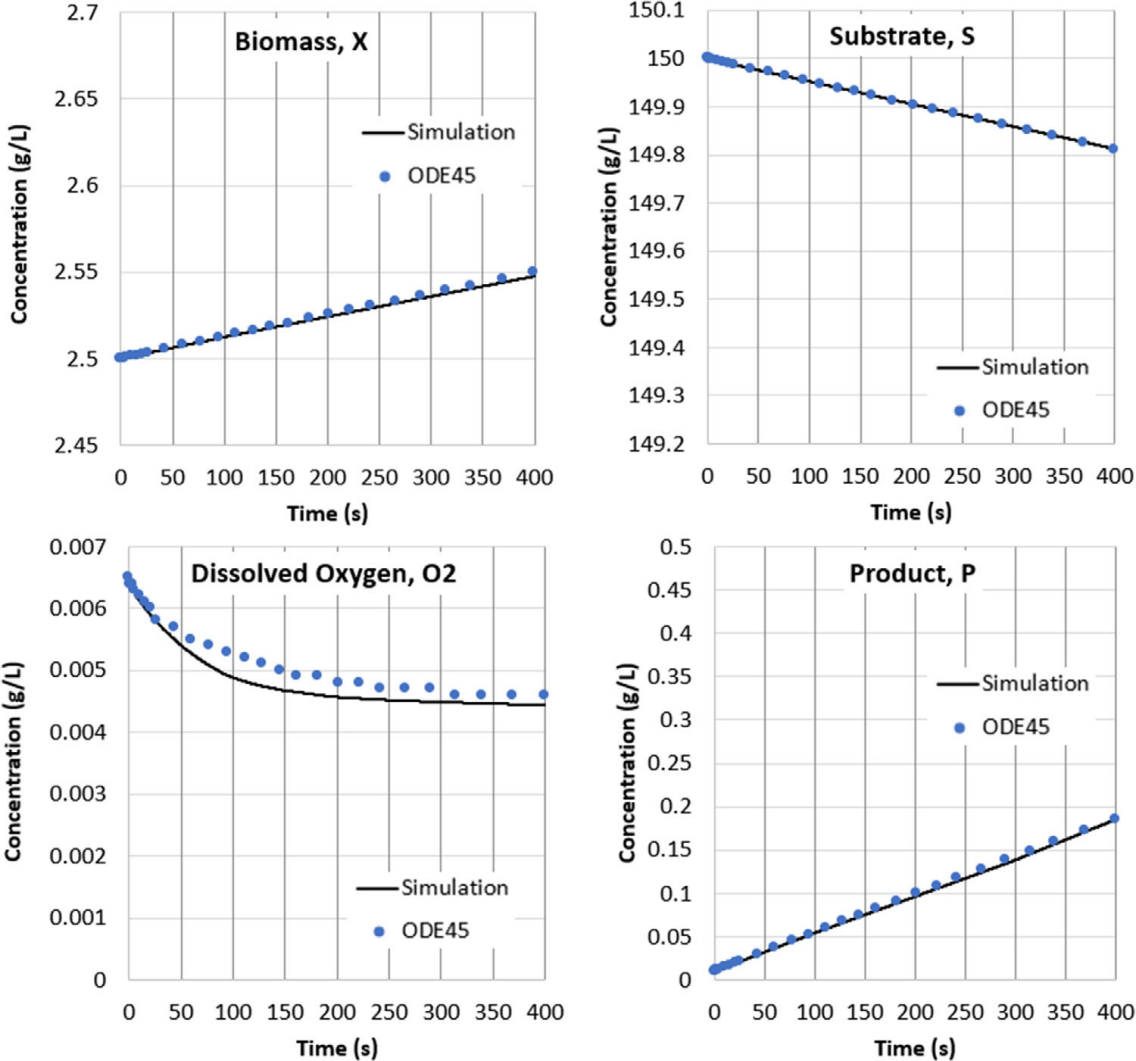
Time evolution of the biomass, substrate, dissolved oxygen, and product (gluconic acid) production during the first 400 s of agitation as predicted from numerical simulation. The initial conditions and reaction parameters are specified in Table 1. Superimposed on these predictions are the numerical solutions to Equations ((15), (16), (17), (18), (19)) using the ODE45 solver in Matlab with a k_l_a of 0.014 1/s.

In Figure 8, we present the time evolution of the biomass, gluconic acid, substrate, and dissolved oxygen concentrations after 20 min of process simulation. We superimpose on these results the solution to Equations ((15), (16), (17), (18), (19)), evaluated using the ODE45 solver in Matlab with the simulation‐predicted oxygen $k_{l}a$ of 0.014 1/s as input to the solution. The reaction rates of the biomass, gluconic acid, and substrate predicted from the simulations are in good agreement with the ODE45 solution. Although the dissolved oxygen concentrations follow similar profiles between the two models, the simulation prediction decreases slightly faster than the numerical solution. This difference is likely caused by the underlying spatial variations in $k_{l}a$ across the fluid. The overall agreement between these results confirms the practicality of incorporating real‐time bioreaction kinetics into fully transient transport modeling simulations.

Note that the ODE45 model assumes that the fluid is well‐stirred and that species concentrations are uniform across the fluid. That is, the biomass, gluconic acid, and substrate reaction time scales are assumed to be order‐of‐magnitude slower than the fluid mixing timescale. The agreement between these fully transient simulations and the ODE45 model corroborates this assumption. This agreement is also in line with predictions from design correlations: at these operating conditions, the blend time in the vessel will be on the order of 10 s [30]. The characteristic consumption rate ($1/\mu_{m}$), however, is on the order of hours.

### 3.4 Modeling longer processes

On a compute node with 8 Nvidia V100 GPUs, simulations advanced at a rate of 1 billion lattice updates per second. As such, the gluconic acid production simulations considered here predicted approximately 1.5 h of ongoing fluid motion/reaction kinetics per day of computer wall time. This runtime includes solving the three‐dimensional and time‐dependent Navier‐Stokes, species transport and species reaction equations. It also includes all bubble tracking, break‐up, and coalescence, as well as the on‐going evaluation of the local fluid viscosity and local fluid body force for each lattice site for each timestep.

The speed of these algorithms, particularly when operating in a GPU environment, represents a step change in the types of questions these simulations can answer. In traditional modeling approaches, short periods of fluid and bubble motion are tracked to predict blending rates, mass transfer rates, and reaction rates during specific periods of a manufacturing process. These instantaneous rates are then interpolated/extrapolated to estimate process outcomes. More novel approaches decompose the problem using stepwise solution strategies that separately resolve the fluid flow, oxygen mass transfer, and bioreactions [22]. With the approach presented here, such extrapolations/interpolations and decompositions are not necessary. Rather, it is practical to model several hour/multiday manufacturing processes while explicitly capturing the ongoing turbulent physics, particle dynamics, and reaction kinetics within the vessel that drive these process outcomes. That is, it becomes practical to directly link evolving system properties to changes in the underlying fluid mechanics/biochemistry.

**FIGURE 9:**
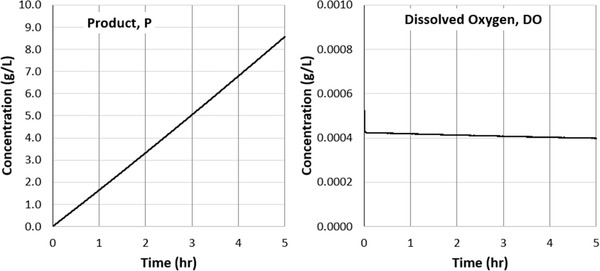
Left: Time‐evolution of gluconic acid (product) growth over 5 h of process simulation. Left: Time‐evolution of dissolved oxygen concentration after 5 h of process simulation.

To illustrate this behavior, in Figure 9 we plot the time evolution of gluconic acid (product) concentration and dissolved oxygen concentration over a 5‐h production period using the operating conditions specified in Table 1. The 5‐h simulation advanced over 225 million timesteps, which was realized after about 3 days of compute time. Since the simulations are explicit, additional hours of simulation time scale linearly with compute time.

**TABLE 1:** Operating conditions and key output parameters, and gluconic acid growth rates from four operating conditions

*Case*	*RPM*	*Gas*	${\dot{Q}}_{g}\left(\mathrm{SLPM}\right)$	$k_{l}a$ *(hr^−^^1^)*	$\left\langle d_{m}\right\rangle$(*mm*)	${\bar{n}}_{b}$	$S_{O_{2}}c_{b,O_{2}}\left(\frac{g}{L}\right)$	$\frac{\mathrm{dP}}{\mathrm{dt}}\left(\frac{g}{L-\mathrm{hr}}\right)$
1	300	Air	8	44.1	1.05	44300	0.0096	1.68
2	300	Air	16	96.1	1.04	93700	0.0096	1.69
3	600	Air	8	154	0.31	970000	0.0096	1.68
4	300	Oxygen	8	44.2	1.03	44200	0.0457	1.99

Abbreviations: RPM, impeller speed; Gas, composition of gas entering through the sparge; ${\dot{Q}}_{g}$, gas flow rate;k_l_a, overall volumetric mass transfer coefficient; $\left\langle d_{m}\right\rangle$, mean bubble diameter during steady‐state operating conditions; ${\bar{n}}_{b}$, time‐average number of bubbles in the system during steady‐state operating conditions; SC_g_, saturation oxygen concentration; $\frac{\mathrm{dP}}{\mathrm{dt}}$, gluconic acid growth rate.

The gluconic acid production rate remains nearly constant over the first 5 h of this process. This trend is consistent with measured data obtained at similar operating conditions over similar durations [9]. The time‐evolution of the dissolved oxygen concentration, however, is more complex and occurs in two distinct phases. During the first phase, which occurs over the first few 100 s of operation, the concentration settles from its initial value to an equilibrium concentration defined by the underlying reactions. This equilibrium timescale is governed by the overall volumetric mass transfer coefficient $k_{l}a$. The second phase begins once the excess dissolved oxygen is removed from the system. During this phase, the ongoing increase in biomass leads to an ongoing increase in the oxygen consumption rate. The net effect of these changes is a decrease in the dissolved oxygen concentration, occurring at a timescale governed by the growth rate μ. Since μ is multiple orders of magnitude smaller than $k_{l}a$, the changes in dissolved oxygen concentration in Phase 2 are slow relative to those in Phase 1. The existence of these two distinct phases is consistent with measured data, which show an initial punctuated drop in oxygen concentration followed by a much slower decrease in the hours of growth that follow [9]. Modelling the physics explicitly allows us to capture both these phases directly.

**FIGURE 10:**
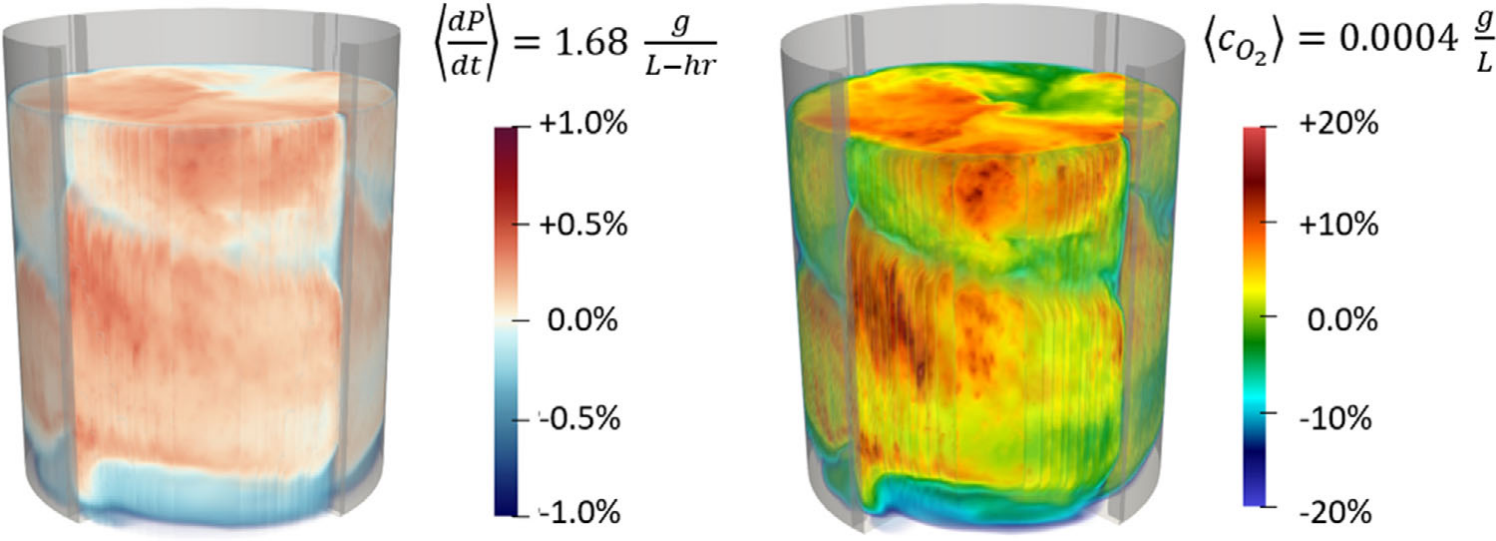
Left: Spatial variation in the instantaneous gluconic acid production rate across the tank following 5 h of simulation. The tank‐average production rate is 1.68 g/L‐hr. The production rate varies by less than +/‐ 1% across the vessel. Right: Spatial variation in the instantaneous dissolved oxygen concentration across the tank following 5 h of simulation. The tank‐average concentration is 0.0004 g/L. The dissolved oxygen concentration varies by more than +/‐ 20% across the vessel.

In Figure 10, we present the spatial variation of the instantaneous gluconic acid production rate across the tank after 5 h of processing. The values here are normalized by the mean production rate in the vessel, as listed in the figure. In this small system, where the blend time is fast relative to the reaction rate, the gluconic acid production rate across the system only varies by ±1%. More variation may be present in vessels with slower blend times or faster reactions. We also present the spatial variation in the instantaneous dissolved oxygen concentration, also taken after 5 h of processing. These values are similarly normalized by the mean dissolved oxygen concentration. The gluconic acid production rates are elevated in regions with the highest concentrations of dissolved oxygen.

### 3.5 Optimizing gluconic acid production

The results presented in Section 3.4 illustrate the practicality of incorporating transient reaction kinetics into the time‐accurate solution to the fluid flow. We now use this model to explore the sensitivity of the process, in terms of optimizing gluconic acid production, to the impeller speed, gas flow rate, and bubble composition.

In Table 1, we compare key output parameters and the gluconic acid growth rate at four unique operating conditions. Note that Case 1 is the base case discussed in Section 3.4. The other three simulation cases included (1) doubling the gas flow rate (2) doubling the impeller speed, and (3) sparging with pure oxygen. For each case, the time‐evolution of the gluconic acid concentration was linear, as shown in Figure 9 for Case 1. The growth rate was therefore calculated from the slope of this concentration profile over the first 2 h of agitation. Although the concentration of the gluconic acid continues to increase beyond this time, measurements suggest that the growth rate remains constant [9].

As expected, doubling the gas flow rate (Case 2 vs. Case 1) approximately doubles the number of bubbles in the system and the overall volumetric mass transfer coefficient. Likewise, doubling the impeller speed (Case 3 vs. Case 1), leads to smaller bubbles, a larger bubble population, and more power dissipation. This combination causes, as expected, a higher volumetric mass transfer coefficient compared to Case 1. However, neither Case 2 nor Case 3 present any meaningful impact on the gluconic acid growth rate. That is, although the $k_{l}a$ in Cases 2 and 3 is 2–4x higher than that of Case 1, the production rate is largely unchanged. The growth rate in Case 4, which was sparged with pure oxygen, is approximately 20% higher than the growth rate in Case 1. This increase was realized despite both cases having similar volumetric mass transfer coefficients. These findings suggest that the product growth rate is linked to the saturation oxygen concentration, but not strongly coupled to the volumetric mass transfer coefficient.

This insensitivity to the volumetric mass transfer coefficient is consistent with expectations from the conservation of mass. Recall from Equation (14) that, in a reacting system, the effects of mass transfer on species concentration are informed by the dimensionless ratio:

(21) $$\xi =\frac{\left(\delta \mu +\phi \right)C_{X}}{S_{O_{2}}c_{b,O_{2}}{\left(k_{l}a\right)}_{O_{2}}}$$

Over the operating conditions and species concentrations considered here, ξ is multiple orders of magnitude lower than 1. Per Equation (15), the equilibrium dissolved oxygen concentration, which informs the product growth rate, will therefore primarily be a function of the oxygen concentration in the bubbles. For Cases 1–3, $c_{o,O_{2}}$ is 0.3 g/L. For Case 4, which is sparged with pure oxygen bubbles, $c_{o,O_{2}}$ is 1.43 g/L. This elevated dissolved oxygen concentration therefore leads to a larger growth rate μ. We recognize that, for gluconic acid, the increase in growth rate may not justify the additional manufacturing costs associated with pure oxygen sparging. The principles discussed here can convey, however, to more valuable processes and products.

## 4 CONCLUSIONS

The purpose of this work was to present a large eddy simulation (LES) based approach to simulate a bioreactor system based on resolution of the transient Navier‐Stokes flow field. This approach is designed to inherently capture the time fluctuations of the species concentrations, subject to imperfect mixing. We used these models to understand mass transport in reacting and non‐reacting systems. The volumetric mass transfer rates were in‐line with measured data for vessels with similar operating conditions. The results also agreed with expectations from first principles. The presence of an ongoing reaction was shown to produce spatially varying species concentrations across the fluid. The magnitude and extent of these variations can be correlated to the reaction rates and the underlying variations in the local volumetric mass transfer coefficient.

Building on these results, we used the model to investigate gluconic acid production by *Aspergillus Niger* in an aerobic fermentation process. The predicted growth rates agreed with experimental observations, confirming the practicality of incorporating real‐time reaction kinetics into fully transient transport modeling frameworks. The speed of these algorithms, particularly when operating in a GPU environment, makes modeling several hour/multiday manufacturing processes practical. That is, it becomes possible in an industrial setting to directly link evolving system properties to changes in the underlying fluid mechanics and chemical reactions. To demonstrate this accessibility, we modeled 5 h of process simulation to observe different regimes of mass transfer and growth kinetics. We then showed how these models could be used to provide mechanistic insights into growth rates and process optimization over several hours of production.

## NOMENCLATURE

**Latin**
*A*
: bubble surface area, m^2^
$A_{T}$
: total bubble surface area, m^2^
*a*
: specific surface area, 1/m
$\vec{a}$
: bubble acceleration, m/s^2^
C: constant
$C_{f}$
: liquid‐phase concentration in the fluid surrounding a bubble, mol/m^3^
$C_{g}$
: gas‐phase concentration in the bubble, mol/m^3^
$C_{s}$
: Smagorinsky coefficient, ‐
*c*
: species concentration, mol/m^3^ or mol/L
Co
: courant number, ‐
*D*
: diffusion coefficient, m^2^/s
*d*
: diameter, m
${\vec{F}}_{a}$
: added mass force on bubble, N
${\vec{F}}_{D}$
: drag force on bubble, N
${\vec{F}}_{f}$
: fluid on fluid voxel, N
${\vec{F}}_{g}$
: net gravity force on bubble, N
${\vec{F}}_{p}$
: pressure force on bubble, N
*H*
: overall mass transfer coefficient, m^3^/s
$k_{L}$
: convective mass transfer coefficient in the fluid surrounding a bubble the bubble, m/s
*m*
: bubble mass, kg
$\dot{n}$
: molar transfer rate, mol/s
*p*
: fluid pressure, Pa
*R*
: reaction rate, mol/s
*S*
: dimensionless gas solubility in the fluid surrounding a bubble, ‐
Sc
: Schmidt number, ‐
*t*
: time, s
*V*
: volume, m^3^
$\vec{V}$
: fluid velocity, m/s

**Greek**
α: dimensionless constant
β: dimensionless constant
ε: specific energy dissipation rate, m^3^/s^2^
ν: fluid kinematic viscosity, m^2^/s
ξ: ratio of oxygen consumption to oxygen transfer, ‐
ρ: fluid density, kg/m^3^
τ: integration time window, s

**Subscripts**
g
: gas phase
i
: bubble
j
: lattice site
k
: species
l
: liquid phase
m
: mean
o
: equilibrium value
r
: reference value

## CONFLICT OF INTEREST

The authors declare no conflict of interest to declare.

## Data Availability

The data that support the findings of this study are available from the corresponding author upon reasonable request.

## References

[1] Mandenius C‐F . *Bioreactors: Design, Operation and Novel Applications*. John Wiley & Sons; 2016.

[2] Lapin A , Muller D , Reuss M . Dynamic behavior of microbial populations in stirred bioreactors simulated with Euler‐Lagrange methods: traveling along the lifelines of single cells. Ind Eng Chem Res. 2004;43:4647‐4656.

[3] Lapin A , Schmid J , Reuss M . Modeling the dynamics of E. coli populations in the three‐dimensional turbulent field of a stirred tank bioreactor: a structured–segregated approach. Chem Eng Sci. 2006;61:4783‐4797.

[4] Gradov DV , Han M , Tervasmäki P , et al. Numerical simulation of biomass growth in OKTOP9000 reactor at industrial scale. Ind Eng Chem Res. 2018;57:13300‐13311. 30416255 10.1021/acs.iecr.8b02765 PMC6219852

[5] Haringa C , et al. Euler‐Lagrange analysis towards representative down‐scaling of a 22m3 Aerobic S. Cerevisiae Fermentation. Chem Eng Sci. 2017;170:653‐669.

[6] Haringa C , Tang W , Wang G , et al. Computational fluid dynamics simulation of an industrial P. Chrysogenum fermentation with a coupled 9‐Pool metabolic model: towards rational scale‐down and design optimization. Chem Eng Sci. 2018;175:12–24.

[7] Zhao Y . Lattice Boltzmann based PDE solver on the GPU. Vis Comput. 2008;24:323‐333.

[8] Haringa C . An analysis of organism lifelines in an industrial bioreactor. Eng Life Sci. 2022:1‐16. 10.1002/elsc.202100159 PMC9815090 36619885

[9] Znad H , Blazej M , Bale V , Markos J . A kinetic model for gluconic acid production by aspergillus niger. Chemical Papers. 2004;58:23‐28.

[10] White FM . Fluid Mechanics. McGraw‐Hill Higher Education, 2002.

[11] Incropera FP , DeWitt DP , Bergman TL , Lavine AS . Fundamentals of Heat and Mass Transfer. 6th ed. John Wiley and Sons; 2007.

[12] Huidan Yu , Sharath S G , Li‐Shi L . DNS and LES of decaying isotropic turbulence with and without frame rotation using lattice Boltzmann method. J Comput Phys. 2005;2:599‐616.

[13] Krüger T , Kusumaatmaja H , Kuzmin A , Shardt O , Silva G , Viggen EM . The Lattice Boltzmann Method: Principles and Practice. Springer International Publishing; 2017.

[14] Thomas J , Sinha K , Shivkumar G , et al. A CFD digital twin to understand miscible fluid blending. AAPS PharmSciTech. 2021;22:91. 33682032 10.1208/s12249-021-01972-5

[15] Thomas JA , Liu X , DeVincentis B , et al. A mechanistic approach for predicting mass transfer in bioreactors. Cheml Eng Sci. 2021:116538.

[16] Farsani HY , Wutz J , DeVincentis B , Thomas JA , Motevalian SP . Modeling mass transfer in stirred microbioreactors. Chem Eng Sci. 2021;248:117146.

[17] Thomas JA , DeVincentis B , Wutz J , Ricci F . Predicting the diameters of droplets produced in turbulent liquid‐liquid dispersion. AIChE Journal. 2021:e17667.

[18] Allen MP , Tildesley DJ . Computer Simulation of Liquids. Oxford University Press; 2017.

[19] Rettinger C , Rüde U . A coupled lattice Boltzmann method and discrete element method for discrete particle simulations of particulate flows. Computers & Fluids. 2018;172:706‐719.

[20] Kawase Y , Moo‐Young M . Mathematical models for design of bioreactors: applications of: kolmogoroff's theory of isotropic turbulence. Chem Eng J. 1990;43:19‐41.

[21] Kawase Y , Halard B , Moo‐Young M . Liquid‐phase mass transfer coefficients in bioreactors. Biotechnol Bioeng. 1992;39:1133‐1140. 18600915 10.1002/bit.260391109

[22] Elqotbi M , Vlaev SD , Montastruc L , Nikov I . CFD modelling of two‐phase stirred bioreaction systems by segregated solution of the Euler–Euler model. Comput Chem Eng. 2013;48:113‐120.

[23] Mohamad AA . Lattice Boltzmann Method. Springer, 2011.

[24] Chiu P‐H , Lin Y‐T . Yan‐Ting. A conservative phase field method for solving incompressible two‐phase flows. J Comput Phys. 2011;230(1):185‐204.

[25] Succi S . *The Lattice Boltzmann Equation: For Fluid Dynamics and Beyond*. Oxford University Press; 2001.

[26] Rathore AS , Sharma C , Persad A . Use of computational fluid dynamics as a tool for establishing process design space for mixing in a bioreactor. Biotechnol Prog. 2012;28:382‐391. 22083975 10.1002/btpr.745

[27] Lide DR . *CRC Handbook of Chemistry and Physics*. CRC press; 2004.

[28] Sander R . Compilation of Henry's law constants (version 4.0) for water as solvent. Atmos Chem Phys. 2015;15:4399‐4981.

[29] M‐Star Simulations. M‐Star CFD User Manual. 2020.

[30] Strand A . *Investigation of Blend Time for Turbulent Newtonian Fluids in Stirred Tanks. Mechanical Engineering*. Rochester Institute of Technology; 2017.

[31] Succi S . *The Lattice Boltzmann Equation: For Fluid Dynamics and Beyond*. Oxford University Press; 2001.

[32] Shu C , Guo Z . *Lattice Boltzmann Method and its Applications in Engineering*. World Scientific, 2013.

[33] Zhu L , He G , Shizhao W , et al. An immersed boundary method based on the lattice Boltzmann approach in three dimensions, with application. Comput Math Appl. 2011;61:3506‐3518.

[34] Pope SB . *Turbulent Flows*. Cambridge University Press; 2000.

[35] M‐Star CFD. 2020.

[36] Hinze JO . Fundamentals of the hydrodynamic mechanism of splitting in dispersion processes. AIChE J. 1955;1:289‐295.

[37] Sungkorn R , Derksen JJ , Khinast JG . Euler–Lagrange modeling of a gas–liquid stirred reactor with consideration of bubble breakage and coalescence. AIChE J. 2012;58:1356‐1370.

[38] Xing C , Wang T , Guo K , Wang J . A unified theoretical model for breakup of bubbles and droplets in turbulent flows. AIChE J. 2015;61:1391‐1403.

[39] Krakau F , Kraume M . Three‐Dimensional observation of single air bubble breakup in a stirred tank. Chem Eng Technol. 2019;42:1357‐1370.

[40] Boshenyatov B . Laws of bubble coalescence and their modeling. J Magnetohydrodyn Plasma Res. 2013;18:311.

[41] Küng VE , Osmanlic F , Markl M , Körner C . Comparison of passive scalar transport models coupled with the Lattice Boltzmann method. Comput Math Appl. 2020;79(1):55‐65.

[42] Leer BV . Towards the ultimate conservative difference scheme. V. A second‐order sequel to Godunov's method. 1, 1979. J Comput Phys. 32:101‐136.

[43] Bird R , Byron S , Warren E , Lightfoot EN . Transport Phenomena. Revised 2nd Edition. John Wiley and Sons, Inc.; 2007.

[44] Ali H , Solsvik J , Wagner JL , Zhang D , Hellgardt K , Park CW . CFD and kinetic‐based modeling to optimize the Sparger design of a large‐scale photobioreactor for scaling up of biofuel production. Biotechnol Bioeng. 2019;116:2200‐2211. 31062867 10.1002/bit.27010

[45] Dano S , Madsen MF , Schmidt H , Cedersund G . Reduction of a biochemical model with preservation of its basic dynamic properties. FEBS J. 2006;273:4862‐4877. 17010168 10.1111/j.1742-4658.2006.05485.x

[46] Davidson KM , Sushil S , Eggleton CD , Marten MR . Using CFD software to estimate circulation time distribution. Biotechnol Prog. 2003;19:1480‐1486. 14524709 10.1021/bp025580d

[47] Douma RD , Verheijen PJT , de Laat WTAM , Heijnen JJ , van Gulik WM . Dynamic gene expression regulation model for growth and penicillin production in Penicillium chrysogenum. Biotechnol Bioeng. 2010;106:608‐618. 20148401 10.1002/bit.22689

[48] Duan X , Feng X , Yang C , Mao Z . CFD Modeling of turbulent reacting flow in a semi‐batch stirred‐tank reactor. Chin J Chem Eng. 2018;26:675–683.

[49] Feng J , Zhang J , Jia W , Yang Y , Liu F , Lin C . An unstructured kinetic model for the improvement of triterpenes production by Ganoderma lucidum G0119 based on nitrogen source effect. Biotechnol Bioproc Eng. 2014;19:727‐732.

[50] Heins AL , Weuster‐Botz D . Population heterogeneity in microbial bioprocesses: origin, analysis, mechanisms, and future perspectives. Bioprocess Biosyst Eng. 2018;41:889‐916. 29541890 10.1007/s00449-018-1922-3

[51] Heins AL , Lundin L , Nunes I , Gernaey KV , Sorensen SJ , Lantz AE . The effect of acetate on population heterogeneity in different cellular characteristics of Escherichia coli in aerobic batch cultures. Biotechnol Prog. 2019;35:e2796. 30816011 10.1002/btpr.2796

[52] Hong HS , Cai ZJ , Li JQ , Shi DS , Wan WQ , Li L . Simulation of gas‐inducing reactor coupled gas‐liquid mass transfer and biochemical reaction. Biochem Engg Jnl. 2014;91:1‐9.

[53] Hristov HV , Mann R , Lossev V , Vlaev SD . A simplified CFD for three‐dimensional analysis of fluid mixing, mass transfer and bioreaction in a fermenter equipped with triple novel geometry impellers. Food Bioprod Process. 2004;82:21‐34.

[54] Wright MR , Bach C , Gernaey KV , Kruhne U , Investigation of the effect of uncertain growth kinetics on a CFD based model for the growth of S. cerevisiae in an industrial bioreactor. Chem Engg Res Des. 2018;144:12‐22.

[55] Wang G , Zhao J , Haringa C , et al. Comparative performance of different scale‐down simulators of substrate gradients in Penicillium chrysogen. Microb Biotechnol. 2018;11:486‐497. 29333753 10.1111/1751-7915.13046 PMC5902331

[56] Takhaveev V , Heinemann M . Metabolic heterogeneity in clonal microbial populations. Curr Opin Microbiol. 2018;45:30‐38. 29477028 10.1016/j.mib.2018.02.004

[57] Sinclair CG , Kristiansen B . Fermentation Kinetic Modeling. Milton Keynes. Open Univ. Press; 1987.

[58] Schwarz MP , PTL K , Verrelli DI , Feng Y . Sequential multi‐scale modelling of mineral processing operations, with application to flotation cells. Miner Eng. 2016;90:2‐16.

[59] Rudniak L , Machniiewski PM , Milewska A , Molga E . CFD modeling of stirred tank chemical reactor: homogenous and heterogeneous reaction systems. Chem Eng Sci. 2004;59:5233‐5239.

[60] Nikolic DD , Frawley PJ . Application of the lagrangian meshfree approach to modelling of batch crystallisation: part I modelling of stirred tank hydrodynamics. Chem Eng Sci. 2016;145:317–328.

[61] Morchain J , Gabelle JC , Cockx A . A coupled population balance model and CFD approach for the simulation of mixing issues in lab‐scale and industrial bioreactors. AIChE J. 2014;1:27–40.

[62] Middleton JC , Smith JM . Chapter 11: gas–liquid mixing in turbulent systems. . [book auth.]. In: Atiemo‐Obeng VA , Kresta SM , Paul EL , eds. Handbook of Industrial Mixing Science and Practice. Wiley; 2004:585‐638.

[63] Buss S . Averaging and Interpolation In 3D Computer Graphics: A Mathematical Introduction with OpenGL. Cambridge University Press; 2003:99‐125.

[64] Thomopoulos NT . Statistical Distributions: Applications and Parameter Estimates. Springer International Publishing, 2017.

[65] Ma Z . Impeller power draw across the full Reynolds number spectrum. MS Thesis, University of Dayton, 2014.

